# Diagnostic concordance and discordance in digital pathology: a systematic review and meta-analysis

**DOI:** 10.1136/jclinpath-2020-206764

**Published:** 2020-09-15

**Authors:** Ayesha S Azam, Islam M Miligy, Peter K-U Kimani, Heeba Maqbool, Katherine Hewitt, Nasir M Rajpoot, David R J Snead

**Affiliations:** 1 Cellular Pathology, University Hospitals Coventry and Warwickshire NHS Trust, Coventry, Coventry, UK; 2 Tissue Image Analytics Laboratory, Department of Computer Science, University of Warwick, Coventry, West Midlands, UK; 3 Nottingham Breast Cancer Research Centre (NBCRC), School of Medicine, University of Nottingham, Nottingham, Nottinghamshire, UK; 4 Warwick Medical School, University of Warwick, Coventry, West Midlands, UK

**Keywords:** pathology, surgical, telepathology, diagnosis, diagnostic techniques and procedures

## Abstract

**Background:**

Digital pathology (DP) has the potential to fundamentally change the way that histopathology is practised, by streamlining the workflow, increasing efficiency, improving diagnostic accuracy and facilitating the platform for implementation of artificial intelligence–based computer-assisted diagnostics. Although the barriers to wider adoption of DP have been multifactorial, limited evidence of reliability has been a significant contributor. A meta-analysis to demonstrate the combined accuracy and reliability of DP is still lacking in the literature.

**Objectives:**

We aimed to review the published literature on the diagnostic use of DP and to synthesise a statistically pooled evidence on safety and reliability of DP for routine diagnosis (primary and secondary) in the context of validation process.

**Methods:**

A comprehensive literature search was conducted through PubMed, Medline, EMBASE, Cochrane Library and Google Scholar for studies published between 2013 and August 2019. The search protocol identified all studies comparing DP with light microscopy (LM) reporting for diagnostic purposes, predominantly including H&E-stained slides. Random-effects meta-analysis was used to pool evidence from the studies.

**Results:**

Twenty-five studies were deemed eligible to be included in the review which examined a total of 10 410 histology samples (average sample size 176). For overall concordance (clinical concordance), the agreement percentage was 98.3% (95% CI 97.4 to 98.9) across 24 studies. A total of 546 major discordances were reported across 25 studies. Over half (57%) of these were related to assessment of nuclear atypia, grading of dysplasia and malignancy. These were followed by challenging diagnoses (26%) and identification of small objects (16%).

**Conclusion:**

The results of this meta-analysis indicate equivalent performance of DP in comparison with LM for routine diagnosis. Furthermore, the results provide valuable information concerning the areas of diagnostic discrepancy which may warrant particular attention in the transition to DP.

## Introduction

Digital pathology (DP), frequently referred to as whole slide imaging (WSI), is a rapidly emerging technology.^1 2^ It has the potential to fundamentally change the way that histopathology is practised by streamlining the workflow,^3^ increasing efficiency,^4 5^ improving diagnostic accuracy and facilitating the platform for the implementation of artificial intelligence–based computer-assisted diagnostics.^2^ Inevitably, these advantages will dictate the adoption of this technology which is very likely to gather pace rapidly in the next few years.

Currently, only a small number of pathology laboratories in the UK and elsewhere are using DP for their routine sign-out purposes.^6^ For more widespread adoption of DP in clinical laboratories, evidence of safety and reliability is needed, ideally in the form of adequately powered multi-site validation studies, demonstrating equivalent performance of DP compared with the existing gold standard of light microscopy (LM).^7^

A number of validation studies have been published to date, but most are small single-site studies and there is considerable variation in the study designs. Previous systematic narrative reviews have summarised the qualitative evidence on the diagnostic reliability of WSI.^8–10^ A systematic review of 38 validation studies between 1999 and 2015 reported an overall diagnostic concordance ranging from 63% to 100%, with a weighted mean of 92.4%.^8^ That review recognised the limitation of small sample size (mean number of cases 140) with variable study design and case types. A subsequent study based on the systematic review of the discordant diagnoses reported 335 discordances (4%) among 8069 comparisons of digital and LM diagnoses. A significant proportion of those discordances were concerning the diagnosis of dysplasia (32%).^9^ Araújo *et al* conducted a systematic review of studies from 2010 to 2017 reporting intra-observer agreement ranging from 87% to 98.3% (κ coefficient range 0.8–0.98).^10^ That review was again based on a small selected series of 13 studies with a mean sample size of 165. Studies comparing the digital diagnosis with either consensus diagnosis or original diagnosis by a different pathologist were not included in this review.

Since the publication of these reviews, larger validation studies have been performed including studies supporting regulatory approval and development of current recommendations by national organisations, providing more guidance and practical advice on the validation process.^11 12^

A meta-analysis to demonstrate the combined accuracy and reliability of DP is still lacking. In the hierarchy of evidence-based healthcare, systematic reviews and meta-analysis using statistical methods to combine the results of individual studies can provide a precise estimate of the effect of size with considerably increased statistical power, placing them at the apex of the evidence pyramid.

The aim of this systematic review and meta-analysis was to review the quantitative evidence across the validation studies, synthesise statistical data and to summarise the evidence on safety and reliability of DP.

## Materials and methods

### Review protocol and registration

This review was conducted in accordance with the guidelines by the Preferred Reporting Items for Systematic Review and Meta-Analyses (PRISMA).^13^ The review protocol was registered with the PROSPERO database (registration number CRD42019145977: Centre for Reviews and Dissemination, University of York, England), the international prospective register of systematic reviews.

### Problem statement

To review the existing literature on the diagnostic (primary and secondary) use of DP and its comparison with LM in the context of the validation process.

- Uncover the strength of the concordance evidence between DP and LM, in order to highlight the usefulness of transition to DP.
- To analyse recent evidence on safety and reliability of DP by identification of discordant diagnosis on the digital platform.

### Definitions

#### Concordance and discordance

Diagnostic concordance was defined as ‘degree of agreement between digital reading and the LM reading for the same sample’. Conversely, any difference or variance between the digital and LM report would reflect discordance. Intra-observer concordance was the preferred method of evaluation, where possible, as per CAP and RCPath guidelines.^11 14^

#### Minor and major discordance

Minor discordance reflects a difference between two reports which is clinically insignificant and would not affect patient management decisions. However, a major discordance leads to or can lead to a difference in clinical decision for patient management.

#### Overall concordance

For this review, the overall clinical concordance was also recorded, which included concordance as well as minor clinically insignificant discordances.

### Search strategy

A literature search was conducted by the primary researcher (ASA) through the key electronic databases: PubMed platform (National Center for Biotechnology Information, U.S. National Library of Medicine, Maryland) including Medline (Medline Industries, Illinois, USA), EMBASE (Elsevier, Amsterdam, The Netherlands), Cochrane Library (London, England) and Google Scholar (Google, California) between 2013 and August 2019. To identify any study being currently undertaken, a search of ClinicalTrials.gov (U.S. National Institutes of Health, Maryland) was performed. A detailed search strategy is available as online supplementary content (online supplementary appendix 1).

10.1136/jclinpath-2020-206764.supp1 Supplementary data

In order to identify any more potentially eligible articles not captured through the aforementioned search, a manual search was conducted via forward citation tracking and reference search of the included studies.

### Article screening and eligibility evaluation

Using Rayyan Qatar Computing Research Institute (QCRI),^15^ all results were screened against a predefined eligibility criterion (table 1) including full abstract and study title by two independent reviewers (ASA and HM).

**Table 1:** Inclusion and exclusion criteria for screening of literature search results

Inclusion criteria	Exclusion criteria
Digital pathology (DP) and light microscopy comparison/validation studies For diagnostic purpose (primary or secondary) Primarily using H&E slides	Studies involving other uses of DP including education, research, molecular or image analysis Predominantly involving immunocytochemistry, special stains, fluorescence or frozen section slides Cytopathology, autopsy, neuropathology Not using whole slide images (telepathology, robotic or static microscopy)

Studies were included provided they met the full inclusion criteria: studies comparing DP with LM reporting for diagnostic purposes, predominantly including H&E-stained slides. Studies were excluded if they explored applications of DP other than diagnosis, predominantly involving ancillary studies or other sub-specialist areas.

The screened results were displayed under one of the following categories: ‘included’, ‘excluded’ and ‘maybe’. The reasons for exclusion were also recorded.

Any disagreements highlighted by Rayyan QCRI between the two reviewers were resolved by discussion. Articles in the ‘maybe’ category were further reviewed by a third reviewer (DRJS). Full texts of all potentially eligible articles were retrieved and reviewed in detail for further evaluation.

### Data extraction

A comprehensive data extraction protocol was developed based on the Cochrane Effective Practice and Organization of Care template.^16^ In addition to the generic domains adapted from the Cochrane—good practice data collection, domains relevant to this review were added. A tailored data record form was designed in Microsoft Excel (V.16.38)

Data extraction was conducted by ASA and supported by other reviewers (DRJS, HM, IMM, KH, NMR). In case of discrepancy between two reviewers, a consensus was reached by discussion. For each included article, the following data items were extracted: study information, participants, interventions, sample, study method, outcome variables and quality assessment (box 1). Corresponding authors of the included studies were contacted to request any further details, where required.

Box 1 **Data collection form with details of domains recorded**

**General Information**

**Participants**

**Interventions**

**Samples**

**Methodology**

**Results**

### Quality assessment (QUADAS 2—tailored)

To assess the quality and risk of bias in individual studies, the Quality Assessment of Diagnostic Accuracy Studies (QUADAS 2)^17^ tool was used. QUADAS 2 was tailored according to this review’s protocol and some of the signalling questions not applicable to the validation studies were excluded. For each of the signalling questions, clear and precise instructions were produced. For each QUADAS domain, the risk of bias was assessed as either ‘low’ or ‘high’ based on the answers to the signalling questions. If insufficient information was provided in the study, the risk of bias was assessed as ‘unclear’.

The quality assessment of all included studies was performed by two reviewers. A review specific tailored QUADAS-2 tool is available as online supplementary content (online supplementary appendix 2).

### Statistical analysis

For each study, we recorded the number of DP–LM comparisons and the number of comparisons where DP and LM diagnoses agreed (number of agreements). We considered two definitions for agreement (concordance): no difference in clinical management (overall concordance) and complete agreement. In three studies,^18–20^ DP–LM comparison for each case was made by multiple pathologists with the number of agreements reported separately for each pathologist. For those studies, we used the average number of agreements for the pathologists.

We considered it reasonable to pool data from all studies but accounted for inherent study-specific characteristics by using random-effects meta-analysis. We took the number of agreements in a study to have a binomial distribution, with a logit link used when modelling the probability of agreement. For studies with 100% agreement, 0.5 was added to the agreements and disagreements. The ‘meta’^21^ package in R^22^ statistical program was used to perform the meta-analysis. A forest plot was used to summarise the pooled results as well as percentage agreements and exact Clopper-Pearson 95% CIs for individual studies.

## Results

### PRISMA flowchart

**Figure 1:**
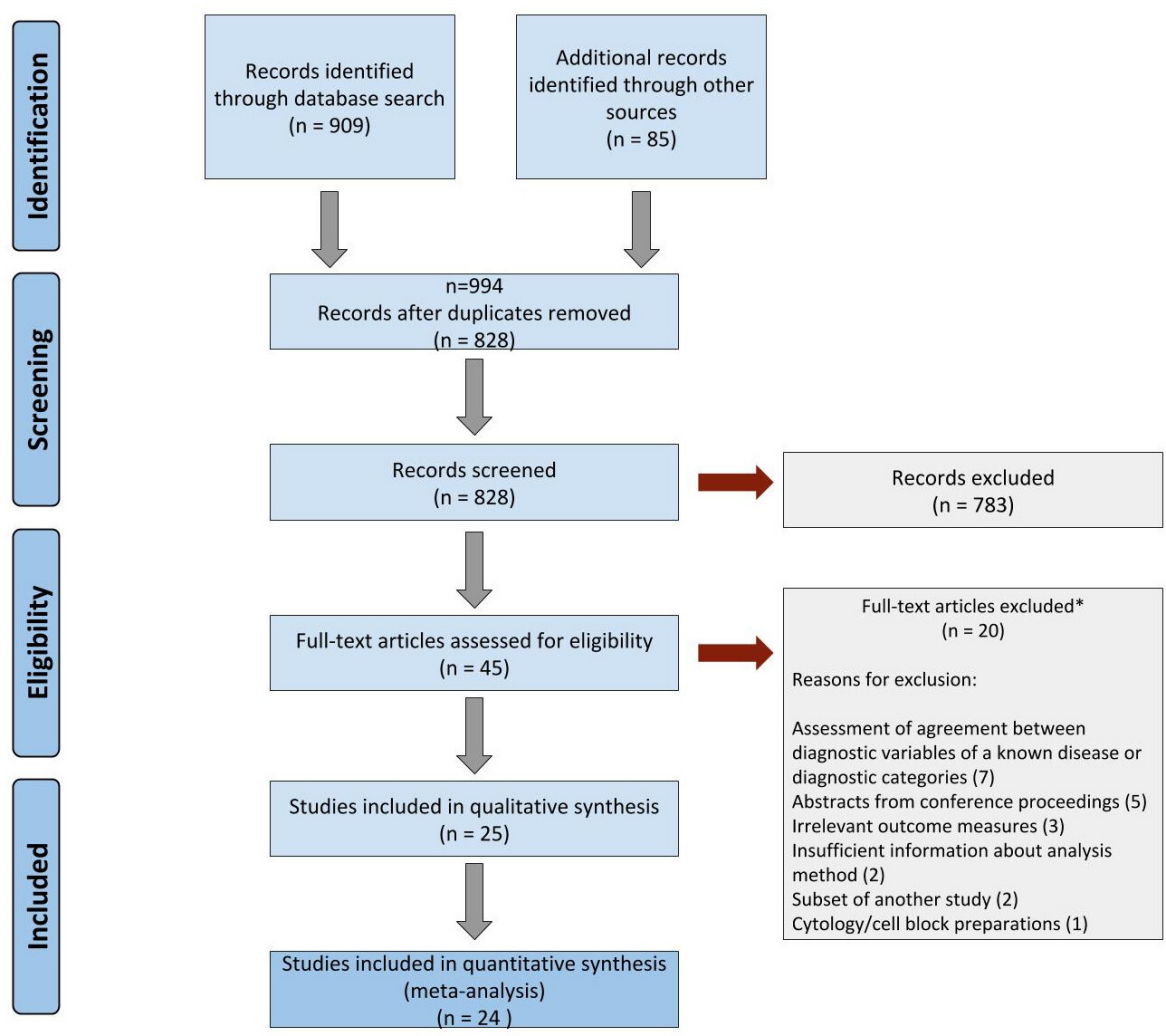
Flowchart following Preferred Reporting Items for Systematic Reviews and Meta-Analyses (PRISMA) guidelines. *Of the 20 articles excluded, 7 assessed agreement between diagnostic variables of a known disease or between broader diagnostic categories, 5 were conference abstracts, 3 included irrelevant outcome measures, 2 stated insufficient about how samples were analysed, 2 were subset of another study and 1 involved cytology cell block preparations.

Figure 1 shows the PRISMA flowchart summarising the results of the review process. The initial systematic search of the literature yielded 994 records in total. After removing the duplicate results, abstracts of 828 records were screened for eligibility.

Eligibility screening identified 45 research studies for full-text review of these articles of which 25 were deemed eligible for inclusion in the review. These studies included a total of 10 410 histology samples with an average sample size of 176. The third reviewer was needed to reach consensus in 8/45 articles. One publication incorporated two distinct study phases with different samples, analysis and results.^23^ The two phases of the same research paper were recorded separately into the total number of included studies. The 25 research papers included in the systematic review were based on the evaluation of total 19 468 LM versus digital comparisons. The quantitative meta-analysis included 24 of these studies.

### Study demographics

Twenty-three studies had been conducted at single centres (92%) and only two (8%) were multi-centre validations. The majority of studies (14; 56%) were from America, 10 (40%) from Europe and 1 (4%) was from Asia. The majority of these studies (56%, n=14) were published from 2015 onward.

### Study characteristics: samples, participants, training, washout time and equipment

**Figure 2:**
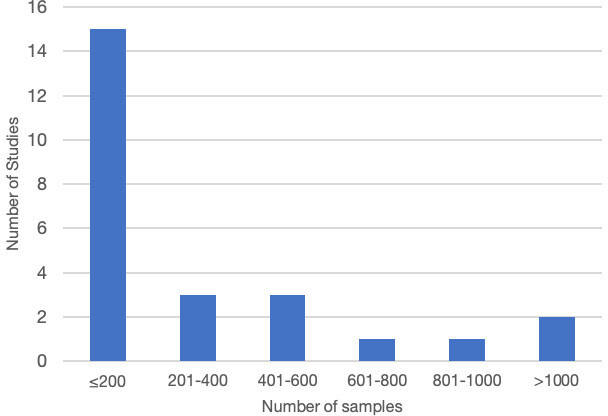
Sample size variations across 25 studies.

**Figure 3:**
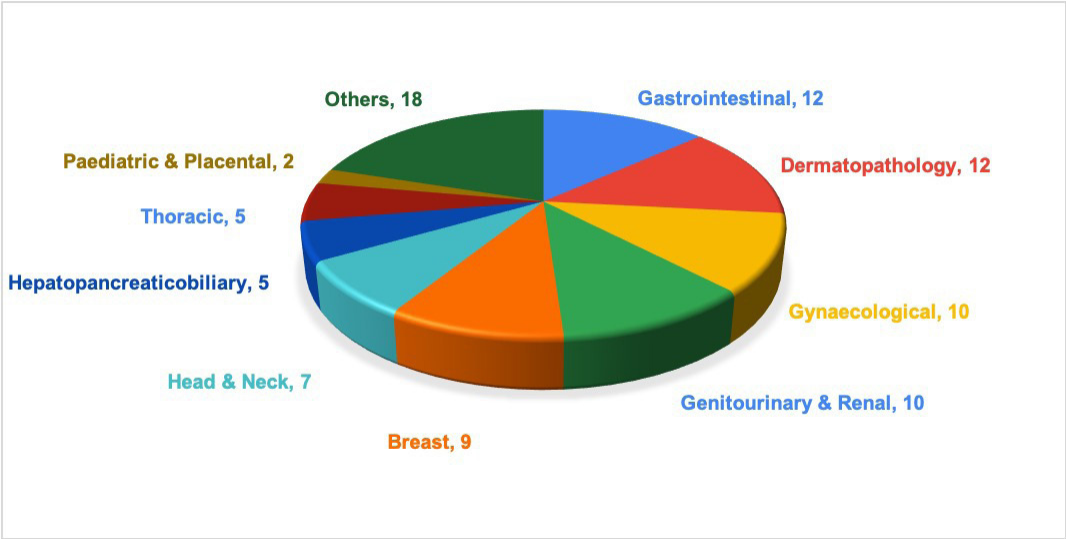
Illustration of the distribution of specialties/organ systems represented across 25 studies (n=number of studies with the inclusion of each organ type).

Sample size (figure 2) varied from 60^24^ to 3017^25^ cases with an average of 176 cases. The majority (15; 60%) examined 200 samples or less with only 3 studies examining 1000 or more cases. The largest sample size reported in validation literature was 3017 to date.^25^ Single specialty samples were selected in 48% (n=12) of the studies, whereas more than one specialty samples were included in 52% (n=13) studies (figure 3). None of the studies stated the inclusion of cancer screening samples.

The number of pathologists who participated in reporting study samples ranged from 1 to 57 with 15 (60%) studies involving fewer than 5 pathologists. In one study, 57 pathologists participated in reporting study samples (n=200) with 28 of them being residents/fellows.^26^ The remaining 23 studies included experienced consultant pathologists while one study did not document the experience level of the participating pathologists.

The participating pathologists were provided training for using the WSI system before reporting the study samples in 13 studies (52%). No training was provided in four studies because the participants had previous experience with using DP for reporting, teaching or tumour boards. Eight studies did not state whether training was provided or not.

Clinical information was provided to the reporting pathologists with each case in the majority of the studies (80%, n=20). Five studies did not state this.

**Figure 4:**
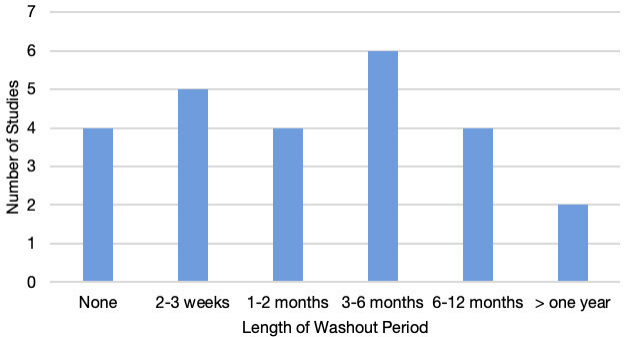
Length of washout period between light microscopy and digital pathology readings.

The length of washout time between DP and LM reporting was variable in the included studies and ranged from 2 weeks to more than a year (figure 4). Four studies (16%) did not use any washout time between the two readings due to the live-validation approach.

The whole slide image scanning devices used in the included studies comprised seven different scanner manufacturers, Aperio (Leica biosystems) being the most commonly used (n=14, 56%). Nine studies had performed slide scanning at ×40 magnification, eight at ×20, six used a combination of ×20 and ×40, and one study used a combination of ×40 and ×60 depending on the sample requirement. Viewing monitor resolution was not commented on in 10 studies. The details of the scanning systems and scan magnification are shown in table 2.

**Table 2:** Various scanning systems and scanning magnifications used in the included studies

	Number of studies	References
**Scanner manufacturers**		
Aperio Scanner (Leica Biosystems)	14	Al-Janabi *et al* 40, Al-Janabi *et al* 41, Araújo *et al* 42, Arnold *et al* 24, Bauer *et al* 43, Bauer and Slaw^3^, Brunelli *et al* 28, Bucks *et al* (1)^23^, Bucks *et al* (2)^23^, Hanna *et al* 44, Kent *et al* 45, Shah *et al* 46, Williams et al^6^, Tabata *et al* 31
Ventana (Roche Diagnostics)	5	Campbell *et al* 18, Ordi *et al* 27, Reyes *et al* 20, Saco *et al* 19, Thrall *et al* 26
Nanozoomer (Hamamatsu)	4	Houghton *et al* 30, Loughrey *et al* 29, Villa *et al* 47, Tabata *et al* 31
Ultra-fast scanner (Philips Intellisite Pathology system)	2	Mukhopadhyay *et al* 32, Araújo *et al* 42
Omnyx VL120 (GE Healthcare)	2	Lee *et al* 48, Snead *et al* 25
Mikroscan vs800 (Olympus Corporation)	1	Tabata *et al* 31
FINO (CLARO, Hirosaki)	1	Tabata *et al* 31
**Scanning magnification**		
×20	9	Al-Janabi *et al* 40, Bauer *et al* 43, Bauer and Slaw^3^, Al-Janabi *et al* 41, Reyes *et al* 20, Ordi *et al* 27, Thrall *et al* 26, Kent *et al* 45, Araújo *et al* 42
×40	8	Houghton *et al* 30, Loughrey *et al* 29, Shah *et al* 46, Saco *et al* 19, Lee *et al* 48, Mukhopadhyay *et al* 32, Villa *et al* 47, Hanna *et al* 44
Mix of ×20 and ×40, depending on specimen type	6	Arnold *et al* 24, Campbell *et al* 18, Bucks *et al* (1)^23^, Bucks *et al* (2)^23^, Tabata *et al* 31, Williams *et al* 6
Mix of ×40 and ×60 (0.137 µm/pixel) depending on specimen type	1	Snead *et al* 25

Only five studies performed a prior sample size calculation by statistical methods. The sample size for three studies^19 25 27^ was based on non-inferiority tests, but with different non-inferiority margins and percentage agreements between DP and LM. Snead *et al* 25 collected the baseline multi-disciplinary team meeting review data to calculate overall intra-observer and inter-observer concordance on ‘LM’ and reached a sample size of just over 3000 cases.

A summary of main characteristics of included studies is available online as online supplementary material (online supplementary appendix 3).

### Diagnostic concordance and discordance

**Figure 5:**
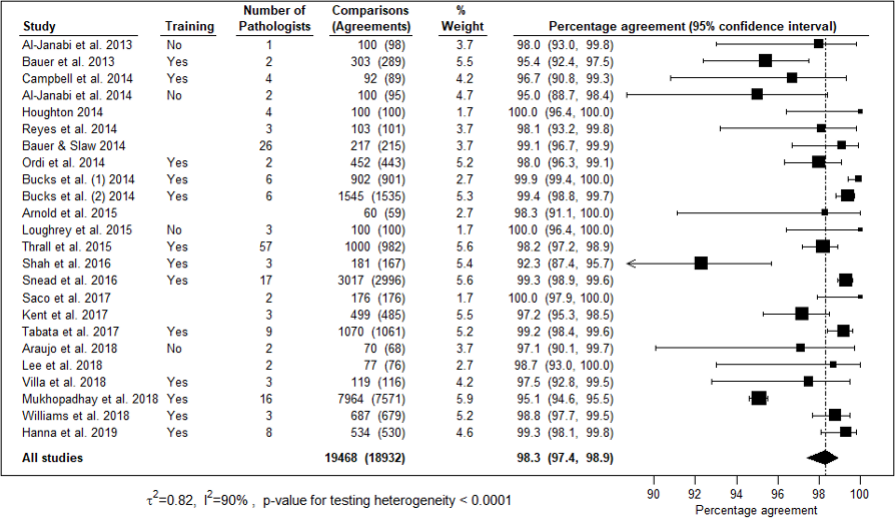
Forest plot representing percentage agreement for overall concordance across 24 studies with the number of comparisons, participating pathologists and digital pathology training.

**Figure 6:**
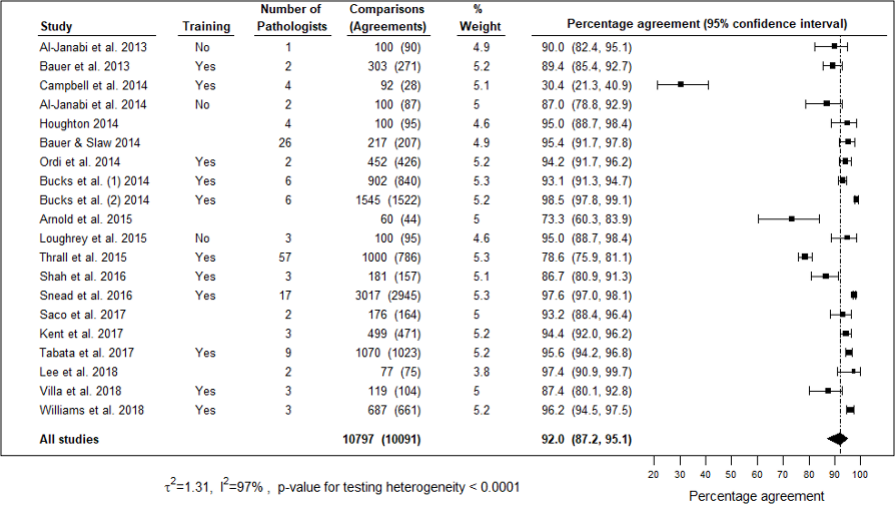
Forest plot representing percentage agreement for complete concordance across 24 studies.

The overall concordance was assessed by measuring complete agreement along with clinically insignificant variations between digital and LM reports. Individual studies percentage agreement across 24 studies included in the meta-analysis ranged from 92.3% to 100%, with the majority (23/24) having percentage agreement above 95% and three studies having 100% agreement (figure 5). The pooled percentage agreement for overall concordance was 98.3% (95% CI 97.4 to 98.9) across 24 studies. One study^28^ used the kappa coefficient (k=0.81) and did not state the concordance percentage. The pooled percentage agreement for complete concordance was 92% (95% CI 87.2 to 95.1) (figure 6). The studies were heterogeneous (I^2^=90%, p<0.0001).

The inter-modality (ie, digital and light microscopy) readings were performed by the same pathologist in 23 (92%) studies. In one study, two-thirds of cases were examined by different pathologists and one-third by the same pathologist.^25^ In one study, the two modalities were examined by different pathologists.^27^

A total of 546 major discordances were reported across 21 studies. Three studies reported only minor differences in results.^19 29 30^ One study^28^ reported the kappa coefficient and did not state the discordance percentage.

Gastrointestinal tract and gynaecological pathology were two most commonly reported specialties among the discordant cases, followed by skin, breast, genitourinary and renal pathology.

### Categorisation of diagnostic discrepancies

Out of 546 major discordances reported across the 25 studies, details of diagnosis and preferred modality were provided for 158 instances. In order to identify and summarise areas of difficulty in the diagnostic performance of WSI, we categorised all the major discordances into four main groups based on the underlying diagnostic discrepancy. Over half (57%) of the reported discordant diagnoses were related to the assessment of nuclear atypia, grading of dysplasia and malignancy (group A). These were followed by challenging diagnoses (26% in group C) and identification of small objects (16% in group B). Each category was further sub-classified into three to seven sub-classifications to capture the nature of discordance. This categorisation was based on the findings of this review and previously reported reviews.^9 10^

Within group A, a total of 68 discordant instances concerning the grading of epithelial dysplasia and nuclear atypia were recorded. The preferred modality for these instances was recorded as LM diagnosis (48/68), digital diagnosis (11/68) and not clearly stated (9/68). Of those cases, where ground truth was LM, the DP under-called (ie, lower grade of dysplasia compared with LM) in 30 of 48 (62.5%) and over-called (higher grade of dysplasia) in 18 of 48 (37.5%).

**Figure 7:**
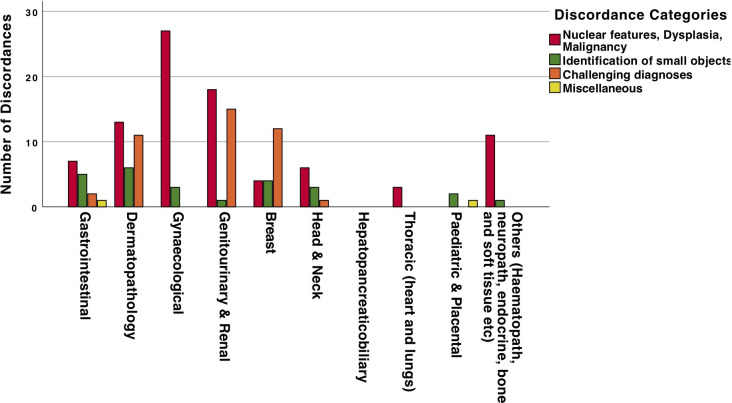
Distribution of four groups of discordances across the specialties involved.

Table 3 shows each category with nature of discordance and organs/sites involved as well as the percentage of all reported major discordances. Figure 7 shows the distribution of four groups of discordances across the specialties involved.

**Table 3:** Categorisation of diagnostic discordances

	Discordance groups (organs involved)	Percentage
A	Nuclear features, dysplasia, malignancy^18 23 25 27 31 32 42–47 49^	57%
	Identification and grading of epithelial dysplasia (colon, stomach, larynx, cervix, lung, penile, bladder and skin) Identification and grading of nuclear atypia (thyroid, uterus, breast and skin) Grading of malignancy (prostate, breast and endocrine pancreas) Missed/over-diagnosis of malignancy (lymph node, thyroid, colon, salivary gland, breast, urethra, testis, lung, prostate, adrenal and kidney) Subtyping of malignancy	
B	Identification of small objects^23–27 31 40 43 48 49^	16%
	Identification of microorganisms, eg, Mycobacteria, fungi, *Helicobacter pylori*, Gram-positive cocci (stomach, oral mucosa, small bowel and skin) Identification of mitotic figures (breast and skin) Identification of inflammatory lesions and cells (oesophagus, colon, duodenum, stomach, cervix, oral mucosa and brain) Identification of granulomata (colon) Detection of metastasis or micro-metastasis (skin, ovary and breast) Identification of Weddellite calcification (breast) Recognition of small area with diagnostic features (endometrium)	
C	Challenging diagnoses^20 25 26 31 32 41 43–49^	26%
	Melanocytic lesions (skin) Atypical breast lesions (eg, B3 lesions) Identification of amyloid and mucin (skin) Focally invasive/malignant lesion (stomach, colon, tongue, breast, thyroid and bladder) Transplant biopsies (kidney)	
D	Miscellaneous^23 25 40^	1%
	Identification of ischaemia, necrosis or granulation tissue (colon) Intestinal metaplasia (stomach) Identification of ganglions (eg, Hirschsprung)	

The remaining underlying reasons for disagreement were stated as follows: difficult case requiring consultation, textural quality of amyloid hard to appreciate on a digital display, lack of clinical information and non-availability of ancillary stains.

### Risk of bias and applicability

The results of quality assessment for risk of bias and applicability in individual studies are displayed in table 4. Across the four domains (case selection, index test, reference standard, flow and timing), the percentage of studies with low risk of bias ranged from 75% (20/25) to 92% (23/25), the percentage of studies with a high risk of bias ranged from 8% to 25%. An unclear risk of bias was found in 12% of the studies. Regarding applicability, all studies showed low concern across case selection domain. Applicability concern was classed as high across the index test domain in one study.

**Table 4:** QUADAS 2—assessment of risk of bias and applicability concerns across 25 studies

Study ID	Risk of bias				Applicability concerns		
	Case selection	Index test	Reference standard	Flow and timing	Case selection	Index test	Reference standard
Al-Janabi *et al* 40	Low	Low	Low	Low	Low	Low	Low
Bauer *et al* 43	Low	Low	Low	High	Low	Low	Low
Campbell *et al* 18	Unclear	Low	Low	Low	Low	Low	Low
Al-Janabi *et al* 41	Low	Low	Low	Low	Low	Low	Low
Brunelli *et al* 28	Unclear	Low	Low	Low	Low	High	Low
Houghton *et al* 30	High	Low	Low	Low	Low	Low	Low
Reyes *et al* 20	Low	Low	Low	Low	Low	Unclear	Unclear
Bauer and Slaw^3^	Low	Low	Low	Low	Low	Low	Low
Ordi *et al* 27	Low	High	High	High	Low	Low	Low
Bucks *et al* (1)^23^	High	Low	Low	Low	Low	Low	Low
Bucks *et al* (2)^23^	High	Unclear	High	Low	Low	Unclear	Unclear
Arnold *et al* 24	Low	Unclear	Low	Low	Low	Unclear	Low
Loughrey *et al* 29	High	Low	Low	Low	Low	Low	low
Thrall *et al* 26	Low	Low	Low	Low	Low	Low	Low
Shah *et al* 46	Low	Low	Low	Low	Low	Low	low
Snead *et al* 25	Low	Low	Low	Low	Low	Low	Low
Saco *et al* 19	Low	Low	Low	Low	Low	Low	Low
Kent *et al* 45	Low	Low	Low	Low	Low	Low	Low
Tabata *et al* 31	High	Low	Low	Low	Low	Low	Low
Araújo *et al* 42	High	Low	Low	Low	Low	Low	Low
Lee *et al* 48	Low	High	Low	High	Low	Unclear	Unclear
Villa *et al* 47	Low	Low	Low	Low	Low	Low	Low
Mukhopadhyay *et al* 32	High	Low	Low	Low	Low	Low	Low
Williams *et al* 6	Low	Unclear	Low	High	Low	Unclear	Low
Hanna *et al* 44	Low	Low	Low	Low	Low	Low	Low

QUADAS2, Quality Assessment of Diagnostic Accuracy Studies.

## Discussion

This is the first meta-analysis of DP studies and largest systematic review to date, based on 25 studies covering 10 412 samples and 19 468 glass versus digital comparisons, including from two recent multi-centre validation studies.^31 32^

These studies demonstrated percentage agreement of 98.3% (95% CI 97.4 to 98.9) for overall concordance and 92% (95% CI 87.2 to 95.1) for complete concordance. This high level of agreement across multiple studies provides strong evidence that DP is a viable alternative to LM for routine practice and, given the multiple additional advantages it offers, can be expected to replace the LM as the main tool for diagnostic histopathology. The studies examined samples from multiple different tissue types suggesting the results are fully representative of the breadth of diagnostic material encountered in routine practice. However, it is noticeable that no studies included ophthalmic samples and too few studies examined renal or paediatric samples to enable meaningful conclusions to be drawn. None of the studies stated which samples, if any, were generated by cancer screening programmes, which has been an area of concern in the UK National Health Service cancer screening programmes (Public Health England personal communication). However, the inclusion of breast, gynaecological and gastrointestinal samples in a large proportion of these studies suggest the results should be relevant to these sample types.

The 546 discordances were analysed to determine the nature of discrepancy and its relevance to patient management. The majority (57%) of clinically significant discordances were related to grading of dysplasia, atypia and malignancy in various tissue types. Grading dysplasia is an important feature which relies largely on subjective assessments, and which is a common source of discrepancy in histopathology.^33–35^ The high incidence of discrepancies in this area may in part reflect this difficulty but also indicates grading dysplasia is an important area to concentrate on in the transition from LM to DP in order to prevent DP introducing an additional error into this challenging diagnostic area. Within these discrepancies, however, there was no consistent pattern towards over-grading of dysplasia with DP as has been suggested in some small studies.^36^

The second the most common discrepancy (26%) concerned challenging diagnoses such as atypical breast lesions (atypical ductal hyperplasia, flat epithelial atypia, low-grade ductal carcinoma in situ), melanocytic lesions, amyloid and small foci of invasive malignancy. Difficulty in locating small objects like micro-organisms, focal inflammation, granulomata, micrometastasis, mitotic figures and Weddellite calcification were encountered in 16% of the discordant cases. These discrepancies all relate diagnostic areas already known to be contentious and where differences of opinion are to be expected^33 35^ irrespective of the modality used to examine the slides.

Some of the diagnostic discrepancies are however likely to be related to DP. These include assessment of objects requiring an appreciation of textural quality, such as deposits of amyloid, mucin and Weddellite calcifications. In these instances, the inability to focus through the ‘z’ plane means the observer using DP is unable to detect the changes in texture normally visible in LM. Second, the recognition of small objects, less than 5 µm in size, such as bacteria has been identified as area of difficulty on DP. Reproduction of these objects in DP systems is clearly inferior to LM because of a combination of loss of detail in the image acquisition, restrictions in the scanning objective and the reliance of a single focal plane. Finally, any object requiring examination under polarised light cannot be adequately catered for in the DP systems currently available. Awareness and knowledge of these issues is essential if pathologists are to understand the limitations of DP and be able to adapt to its use in their own practice safely.

Across this meta-analysis, there are potentially important variations in study design including sample selection criteria, equipment used and length of washout time that could influence the results. Where possible, these potential sources of bias have been assessed using the QUADAS 2 tool, which demonstrated a low risk of bias for the majority of studies. As expected with an emerging new technology, this meta-analysis has captured results from studies using differing technologies. Despite improvements in image quality, the more recent equipment can provide there was no difference in discrepancy rates over the course of these studies. This most likely indicates that even the earlier DP studies used equipment capable of delivering a diagnostic tool equivalent to LM.

Although this review provides strong and consistent evidence of the equivalent performance of DP in comparison with LM, it also highlights certain areas where the evidence is still weak. First, the majority of the studies provide no evidence base for sample size and no power calculation, which prevent a statistical measurement of inferiority. Second, the intra-observer and inter-observer variability on existing LM platform is unknown, so it is not possible to calculate the number of discrepancies actually related to viewing modality.

## Conclusion

Although the barriers to wider adoption of DP have been multifactorial, limited evidence of reliability has been a significant contributor. The COVID-19 pandemic acted as a catalyst to change working practice across the health sector.^37^ The flexibility provided by digitising the workflow is fundamental to these changes, and DP is a key step to enabling this to happen in cellular pathology.^38 39^ The demand for widespread adoption of DP is growing further as a result.

The results of this meta-analysis represent significant evidence to indicate the equivalent performance of DP for routine diagnosis. Furthermore, the categorisation of diagnostic discordances highlights a number of potential limitations, where alternative solutions may be needed. The findings also indicate how the enrichment of sample selection in future studies may improve the evidence base further.

**Take home messages**

**General Information**

**Participants**

**Interventions**

**Samples**

**Methodology**

**Results**
