# Supplementary material for: Diagnostic concordance and discordance in digital pathology: a systematic review and meta-analysis
Source: J Clin Pathol. 2020 Sep 15;74(7):448–55. doi: 10.1136/jclinpath-2020-206764 (PMC8223673; doi:10.1136/jclinpath-2020-206764)
Supplement: Supplementary data [file jclinpath-2020-206764supp001.pdf]

Supplementary material

Appendix 1

Search strategy

For PubMed/Medline, EMBASE and Cochrane

The database search used the following terms:

Digital pathology OR Whole slide imag* OR Virtual microscopy OR Digital microscopy OR Digital slides OR Virtual slides OR Telepathology OR Tele-microscopy

AND

Light microscopy OR Conventional microscopy OR Traditional microscopy OR glass slides

AND

Diagnosis OR Validation OR Comparison OR Concordance OR Accuracy

Limits applied were:

- Language (English)
- Human studies
- Between 2013 and 2017

Alternate terms digitized slide and whole slide scanner were also used.
The asterisk character (*) was used to match more than one word ending.

Appendix 2

Main characteristics of the included studies

Study	Number of cases	Number of pathologists	Organ Systems	Washout Period	Number of DP vs LM Comparisons (Agreement/concordance)	Overall Concordance (percentage)
Al-Janabi et al. 2013	100	1	Paediatric and placental	More than 1 year	100(98)	98
Bauer et al. 2013	303	2	Mix	1 year, at least	303(289)	95.4
Campbell et al. 2014	85	4	Breast	6 to 10 months	92(89)	96.7
Al-Janabi et al. 2014	100	2	Genitourinary & Renal	6 months to one year, at least	100(95)	95
Brunelli et al. 2014	61	2	Mix	2 weeks		
Houghton et al. 2015	100	4	Mix	Mean 1.8 years	100(100)	100
Reyes et al. 2014	103	3	Breast	2-3 weeks	103(101)	98.1
Bauer & Slaw 2014	217	26	Mix	47 days (average)	217(215)	99.1
Ordi et al. 2014	452	2	Gynae	None	452(443)	98
Bucks et al. (1) 2014	60	6	Mix	3-6 months	902(901)	99.9
Bucks et al. (2) 2014	524	6	GI	None	1545(1535)	99.4
Arnold et al. 2015	60	Not stated	Paediatric and placental	3 months to 6 years	60(59)	98.3
Loughrey et al. 2015	100	3	GI	6 months, at least	100(100)	100
Thrall et al. 2015	200	57	Mix	3 weeks, at least	1000(982)	98.2
Shah et al. 2016	181	3	Skin	8 weeks	181(167)	92.3
Snead et al. 2016	3017	17	Mix	3 weeks	3017(2996)	99.3
Saco et al. 2017	176	2	Genitourinary & Renal	1.5 to 4 months, at least	176(176)	100
Kent et al. 2017	499	3	Skin	Minimum 30 days	499(485	97.2
Tabata et al. 2017	900	9	Mix	>2 weeks	1070(1061)	99.2
Araujo et al. 2018	70	2	Head and Neck	3 months	70(68)	97.1

Lee et al. 2018	93	2	Skin	None	77(76)	98.7*
Villa et al. 2018	119	3	Mix	5.4 months	119(116)	97.5
Mukhopadhay et al. 2018	1992	16	Mix	4 weeks, at least	7964(7571)	95.1
Williams et al. 2018	694	3	Breast	None	687(679)	98.8
Hanna et al. 2019	204	8	Mix	13 weeks	534(530)	99.3

Appendix 3

Tailored QUADAS2 TOOL – A quality assessment tool for diagnostic accuracy studies.

Introduction to QUADAS2

QUADAS-2 is designed to assess the quality of diagnostic accuracy studies.

It consists of four key domains covering

1. Sample selection
2. Index test (DP)
3. Reference test (LM)
4. Flow and timing

Each domain is assessed in terms of the *risk of bias* and in terms of *concerns regarding applicability*. To help reach a judgement on the risk of bias, *signalling questions* are included. These flag aspects of study design related to the potential for bias and aim to help reviewers make risk of bias judgements. They are answered as "yes", "no", or "unclear", and are phrased such that "yes" indicates low risk of bias.

Risk of bias is judged as "low", "high", or "unclear".

Domain 1: Sample Selection	
Signalling question 1: Was a consecutive or random sample of patients enrolled?	Yes/No/Unclear
Signalling question 2: Did the study avoid inappropriate exclusions?	Yes/No/Unclear
A: RISK OF BIAS (based on responses to above signalling questions)<br>Could the selection of patients have introduced bias?	RISK:<br>LOW/HIGH/UNCLEAR
B CONCERNS REGARDING APPLICABILITY<br>Is there concern that the included patients do not match the review question?	RISK:<br>LOW/HIGH/UNCLEAR
Domain 2: Index test (Digital reading)	
Signalling question 1: Were the index test results interpreted without knowledge of the results of the reference standard?	Yes/No/Unclear
A: RISK OF BIAS (based on response to the above signalling question)<br>Could the conduct or interpretation of the index test have introduced bias?	RISK:<br>LOW/HIGH/UNCLEAR
B CONCERNS REGARDING APPLICABILITY<br>Is there concern that index test, its conduct, or interpretation differ from review question?	RISK:<br>LOW/HIGH/UNCLEAR
Domain 3: Reference test (glass reading)	
Signalling question 1: Were the reference test results interpreted without knowledge of the results of the reference standard?	Yes/No/Unclear
A: RISK OF BIAS (based on answers to the above signalling questions)<br>Could the conduct or interpretation of the reference test have introduced bias?	RISK:<br>LOW/HIGH/UNCLEAR
B CONCERNS REGARDING APPLICABILITY<br>Is there concern that reference test, its conduct, or interpretation differ from review question?	RISK:<br>LOW/HIGH/UNCLEAR
Domain 4: Flow and timing	
Signalling question 1. Was there an appropriate interval between index test(s) and reference standard?	Yes/No/Unclear
Signalling question 2. Did all patients receive a reference standard?	Yes/No/Unclear
Signalling question 3. Did patients receive the same reference standard?	Yes/No/Unclear
Signalling question 4. Were all patients included in the analysis?	Yes/No/Unclear
RISK OF BIAS (based on answers to above questions)<br>Could the patient flow have introduced bias?	RISK:<br>LOW/HIGH/UNCLEAR

The tool will be completed in four phases:

1) state the review question
2) develop review specific guidance
3) review the published flow diagram for the primary study
4) judgement of bias and applicability.

Review authors need to use the guidelines developed in phase 2 to judge risk of bias.

If all signalling questions for a domain are answered “yes” then risk of bias can be judged “low”. If any signalling question is answered “no” this flags the potential for bias. The “unclear” category should be used only when insufficient data are reported to permit a judgment.

2. Review specific guidance

These guidance notes are based on QUADAS2 Guidelines, with few explanations specific to this systematic review.

These guidance notes will help the reviewer to assess the ‘risk of bias’ and ‘concerns regarding applicability’, based on the answers to the signalling questions.

Domain 1: Sample Selection

Justification/Support for assessing the risk of bias

What are inappropriate exclusions?
Exclusion of difficult, complex cases, larger samples, excision samples or cases where consensus could not be reached on diagnosis for the study.

What are appropriate exclusions?
Missing slides (due to any reason) or broken slides. Only if reason has been stated clearly.

Sample selection criteria

QUADAS recommends consecutive or random selection ideally. Consecutive selection is most likely to represent routine case mix in a lab. A smaller number of selected cases can be used, only if reasons have been stated clearly. A random selection of cases is associated with less risk of bias, as the selection is not controlled.

If a study includes predominantly selected cases (for various reasons), then it is likely to introduce selection bias.
The underlying reasons could be; know diagnosis, known sample type, known difficulty level.

Risk of bias is judged as “low”, “high”, or “unclear”

Low risk	If all signalling questions are answered “yes” then risk of bias can be judged “low”<br>OR<br>If signalling question 1 is answered “yes” and signalling question 2 “unclear then risk of bias can be judged as “low” provided that the study did not exclude >10% of samples as inappropriate exclusions.
High risk	If any signalling question is answered “no” then risk of bias can be judged “high”.
Unclear risk	If all signalling questions are answered “unclear” then risk of bias can be judged “unclear”.

Domain 2: Index test

Justification/Support for assessing the risk of bias.

Reporting pathologists should be blinded to the original or reference diagnosis, otherwise it has potential for bias.

Risk of bias is judged as “low”, “high”, or “unclear”

Low risk	If signalling question is answered “yes” then risk of bias can be judged “low”
High risk	If signalling question is answered “no” then risk of bias can be judged “high”.

Unclear risk	If signalling question is answered "unclear" then risk of bias can be judged "unclear".

Domain 3: Reference test

Justification/Support for assessing the risk of bias.

Reporting pathologists should be blinded to the original or index (DP) diagnosis, otherwise it has potential for bias.

Risk of bias is judged as "low", "high", or "unclear"

Low risk	If signalling question is answered "yes" then risk of bias can be judged "low"
High risk	If signalling question is answered "no" then risk of bias can be judged "high".
Unclear risk	If signalling question is answered "unclear" then risk of bias can be judged "unclear".

Domain 4: Flow and timing

Justification/Support for assessing the risk of bias:

Based on CAP guidelines, a minimum of 2 weeks washout interval is required to minimize the recall bias. If a study uses live validation method, then this flags the potential risk of bias.

Risk of bias is judged as "low", "high", or "unclear"

Low risk	If all signalling questions are answered "yes" then risk of bias can be judged "low"
High risk	If any signalling question is answered "no" then risk of bias can be judged "high".
Unclear risk	If more than two signalling questions are answered "unclear" then risk of bias can be judged "unclear".

Once completed, the results of risk of bias and applicability (in blue font) will be represented in a table.
